# Supplementary material for: Multifactorial processes underlie parallel opsin loss in neotropical bats
Source: eLife. 2018 Dec 18;7:e37412. doi: 10.7554/eLife.37412 (PMC6333445; doi:10.7554/eLife.37412)
Supplement: Supplementary file 2. — Grey boxes indicate the preferred model inferred from a likelihood ratio test. lnL: log-likelihood; np: number of parameters; TL: tree length; k: kappa (transition/transversion ratios); LR: likelihood ratio; p: p-value of likelihood ratio of alternative relative to null for each test [file elife-37412-supp2.docx]

**Supplementary file 2**

Results of molecular evolution branch analyses for each of the three opsin genes tested for differences in rates of nonsynonymous to synonymous substitutions (*ω*) for lineages that lack the S-cone and lineages that have retained the S-cone. Grey boxes indicate the preferred model inferred from a likelihood ratio test. *lnL*: log-likelihood; *np*: number of parameters; *TL*: tree length; *k*: kappa (transition/transversion ratios); LR: likelihood ratio; *p*: *p*-value of likelihood ratio of alternative relative to null for each test

| **Gene** | **Model** | **lnL** | **np** | **TL** | ***k*** | **LR** | ***p*** | **ω** |
| --- | --- | --- | --- | --- | --- | --- | --- | --- |
| ***OPN1SW*** | *null* | -5692.64 | 61 | 2.6 | 4.6 | --- | --- |  |
|  |  |  |  |  |  | *ω*_background_ | | 0.21 |
|  | *2 branch* | -5669.08 | 62 | 2.6 | 4.7 | 47.12 | 6.68e-12 |  |
|  |  |  |  |  |  | *ω*_background_ | | 0.13 |
|  |  |  |  |  |  | *ω*_S-cone.absent_ | | 0.36 |
|  | *3 branch* | -5657.14 | 63 | 2.6 | 4.7 | 70.99 | 3.84e-16 |  |
|  |  |  |  |  |  | *ω*_background_ | | 0.13 |
|  |  |  |  |  |  | *ω*_OPN1SW.intact_ | | 0.24 |
|  |  |  |  |  |  | *ω*_OPN1SW.pseudo_ | | 0.78 |
| ***OPN1LW*** | *null* | -4194.92 | 65 | 1.5 | 6.6 | --- | --- |  |
|  |  |  |  |  |  | ω_background_ | | 0.10 |
|  | *2 branch* | -4193.44 | 66 | 1.5 | 6.6 | 2.95 | 0.09 |  |
|  |  |  |  |  |  | ω_background_ | | 0.08 |
|  |  |  |  |  |  | ω_S-cone.absent_ | | 0.12 |
|  | *3 branch* | -4190.33 | 67 | 1.5 | 6.6 | 9.18 | 0.01 |  |
|  |  |  |  |  |  | ω_background_ | | 0.08 |
|  |  |  |  |  |  | *ω*_OPN1SW.intact_ | | 0.08 |
|  |  |  |  |  |  | *ω*_OPN1SW.pseudo_ | | 0.19 |
| ***RHO*** | *null* | 4233.05 | 65 | 1.9 | 7.8 | --- | --- |  |
|  |  |  |  |  |  | ω_background_ | | 0.03 |
|  | *2 branch* | -4232.88 | 66 | 1.9 | 7.8 | 0.34 | 0.56 |  |
|  |  |  |  |  |  | ω_background_ | | 0.03 |
|  |  |  |  |  |  | ω_S-cone.absent_ | | 0.03 |
|  | *3 branch* | -4232.73 | 67 | 1.9 | 7.8 | 0.63 | 0.73 |  |
|  |  |  |  |  |  | ω_background_ | | 0.03 |
|  |  |  |  |  |  | *ω*_OPN1SW.intact_ | | 0.03 |
|  |  |  |  |  |  | *ω*_OPN1SW.pseudo_ | | 0.03 |

**Table S3**

Results of molecular evolution branch analyses for each opsin gene that test for differences in rates of nonsynonymous to synonymous substitutions (*ω*) for frugivorous and non-frugivorous lineages. Grey boxes indicate the preferred model inferred from a likelihood ratio test. *lnL*: log-likelihood; *np*: number of parameters; *TL*: tree length; *k*: kappa (transition/transversion ratios); LR: likelihood ratio; *p*: *p*-value of likelihood ratio of alternative relative to null for each test

| **Gene** | **Model** | **lnL** | **np** | **TL** | ***k*** | **LR** | ***p*** | **ω** | |
| --- | --- | --- | --- | --- | --- | --- | --- | --- | --- |
| ***OPN1SW*** | *null* | -6355.96 | 77 | 2.9 | 4.5 | --- | --- |  | |
|  |  |  |  |  |  | *ω*_background_ | | | 0.20 |
|  | *alternative* | -6355.52 | 78 | 2.9 | 4.5 | 0.88 | 0.35 |  | |
|  |  |  |  |  |  | *ω*_background_ | | | 0.21 |
|  |  |  |  |  |  | *ω*_frugivore_ | | | 0.18 |
| ***OPN1LW*** | *null* | -4662.16 | 81 | 1.7 | 6.9 | --- | --- |  | |
|  |  |  |  |  |  | ω_background_ | | | 0.10 |
|  | *alternative* | -4661.97 | 82 | 1.7 | 6.9 | 0.38 | 0.54 |  | |
|  |  |  |  |  |  | ω_background_ | | | 0.10 |
|  |  |  |  |  |  | *ω*_frugivore_ | | | 0.09 |
| ***RHO*** | *null* | -4665.92 | 81 | 2.1 | 7.7 | --- | --- |  | |
|  |  |  |  |  |  | ω_background_ | | | 0.03 |
|  | *alternative* | -4659.04 | 82 | 2.1 | 7.7 | 13.77 | 2.07e-04 |  | |
|  |  |  |  |  |  | ω_background_ | | | 0.04 |
|  |  |  |  |  |  | *ω*_frugivore_ | | | 0.01 |

**Table S4**

Cone densities in 14 species representative of different diet types. Mean cones densities across the retinal tiles quantified in individual flat mounted retinas. Densities were estimated after averaging the count for each tile.

| **Species** | **L-opsin** | **S-opsin** | **Field number** |
| --- | --- | --- | --- |
| *Artibeus jamaicensis* | 5534 | 2688 | 2015-071 |
| *Artibeus jamaicensis* | 5587 | 2253 | 2016-040 |
| *Artibeus phaeotis* | 5557 | 3642 | 2016-010 |
| *Artibeus phaeotis* | 6539 | 3841 | 2016-007 |
| *Carollia sowelli* | 4417 | 1094 | 2015-131 |
| *Carollia sowelli* | 4769 | 1162 | 2015-113 |
| *Carollia sowelli* | 4706 | 1178 | 2015-113 |
| *Carollia sowelli* | 4672 | 1194 | 2015-120 |
| *Sturnira lilium* | 4918 | 952 | 2015-123 |
| *Sturnira lilium* | 5109 | 1015 | 2015-127 |
| *Monophyllus redmani* | 2557 | 0 | 2015-027 |
| *Monophyllus redmani* | 2659 | 0 | 2015-020 |
| *Monophyllus redmani* | 2691 | 0 | 2015-020 |
| *Erophylla sezekorni* | 3681 | 0 | 2016-025 |
| *Erophylla sezekorni* | 3974 | 0 | 2016-007 |
| *Erophylla sezekorni* | 3499 | 0 | 2016-007 |
| *Erophylla sezekorni* | 3674 | 0 | 2016-027 |
| *Glossophaga soricina* | 5583 | 328 | 2015-153 |
| *Glossophaga soricina* | 5020 | 350 | 2015-118 |
| *Brachyphylla nana pumila* | 5471 | 0 | DR231 |
| *Brachyphylla nana pumila* | 5481 | 0 | DR231 |
| *Desmodus rotundus* | 3406 | 0 | 2015-150 |
| *Desmodus rotundus* | 3986 | 0 | 2015-126 |
| *Desmodus rotundus* | 3980 | 0 | 2015-145 |
| *Pteronotus quadridens* | 7227 | 4263 | 2016-109 |
| *Pteronotus quadridens* | 8100 | 5022 | 2016-108 |
| *Pteronotus quadridens* | 8109 | 6652 | 2016-119 |
| *Pteronotus quadridens* | 6487 | 7053 | 2016-121 |
| *Mormoops blainvillei* | 4089 | 0 | 2016-142 |
| *Mormoops blainvillei* | 3869 | 0 | 2016-142 |
| *Macrotus waterhousii* | 6692 | 0 | DR194 |
| *Gardnerycteris crenulatum* | 2080 | 0 | 2015-129 |
| *Phyllops falcatus* | 5944 | 2716 | DR135 |

**Table S5**

Summary of Bayesian logistic regression models of the presence of an *OPN1SW* ORF as a function of diet or cave roosting. All per-tree estimated sample sizes exceeded 200. *R^2^*, variance explained by sample-wide factors; *Σ*, species-specific phylogenetic effect.

| **formula** | ***R^2^*** | **parameter** | **median** | **2.5%** | **97.5%** |
| --- | --- | --- | --- | --- | --- |
| Presence*_i_* ~ *a* + *b* . plant_prevalent*_i_* + *Σ* | 0.038 | intercept (*a*) | 2.090 | 0.359 | 5.288 |
|  |  | *b* | 0.811 | -1.458 | 3.368 |
|  |  | *Σ* | 2.891 | 0.036 | 10.86 |
| Presence*_i_* ~ *a* + *b* . fruit_prevalent*_i_* + *Σ* | 0.078 | intercept (*a*) | 1.971 | 0.356 | 5.103 |
|  |  | *b* | 1.139 | -0.896 | 3.515 |
|  |  | *Σ* | 2.527 | 0.022 | 10.34 |
| Presence*_i_* ~ *a* + *b* . insect_prevalent*_i_* + *Σ* | 0.068 | intercept *(a)* | 1.675 | -0.643 | 4.980 |
|  |  | *b* | 1.079 | -1.065 | 3.965 |
|  |  | *Σ* | 3.094 | 0.050 | 11.122 |
| Presence*_i_* ~ *a* + *b* . cave_roost*_i_* + *Σ* | 0.106 | intercept *(a)* | 3.369 | 1.048 | 7.658 |
|  |  | *b* | -1.366 | -4.260 | 0.723 |
|  |  | *Σ* | 2.589 | 0.020 | 10.515 |

**Table S6**

Summary of Bayesian logistic regression models of the presence of an *OPN1SW* mRNA as a function of diet or cave roosting. All per-tree estimated sample sizes exceeded 200. *R^2^*, variance explained by sample-wide factors; *Σ*, species-specific phylogenetic effect.

| **formula** | ***R^2^*** | **parameter** | **median** | **2.5%** | **97.5%** |
| --- | --- | --- | --- | --- | --- |
| Presence*_i_* ~ *a* + *b* . plant_prevalent*_i_* + *Σ* | 0.027 | intercept (*a*) | 1.499 | 0.101 | 3.668 |
|  |  | *b* | 0.580 | -1.713 | 2.647 |
|  |  | *Σ* | 0.987 | 0.002 | 7.728 |
| Presence*_i_* ~ *a* + *b* . fruit_prevalent*_i_* + *Σ* | 0.610 | intercept (*a*) | 1.082 | -0.040 | 2.593 |
|  |  | *b* | 3.274 | 0.796 | 7.428 |
|  |  | *Σ* | 0.459 | 0.001 | 5.044 |
| Presence*_i_* ~ *a* + *b* . insect_prevalent*_i_* + *Σ* | 0.029 | intercept *(a)* | 2.069 | 0.197 | 4.510 |
|  |  | *b* | -0.589 | -2.568 | 1.551 |
|  |  | *Σ* | 0.952 | 0.002 | 7.606 |
| Presence*_i_* ~ *a* + *b* . cave_roost*_i_* + *Σ* | 0.292 | intercept *(a)* | 3.135 | 1.002 | 6.121 |
|  |  | *b* | -1.863 | -4.832 | 0.271 |
|  |  | *Σ* | 0.568 | 0.001 | 5.564 |

**Table S7**

Summary of Bayesian logistic regression models of the presence of S-cones as a function of diet or cave roosting. All per-tree estimated sample sizes exceeded 200. *R^2^*, variance explained by sample-wide factors; *Σ*, species-specific phylogenetic effect.

| **formula** | ***R^2^*** | **parameter** | **median** | **2.5%** | **97.5%** |
| --- | --- | --- | --- | --- | --- |
| Presence*_i_* ~ *a* + *b* . plant_prevalent*_i_* + *Σ* | 0.421 | intercept (*a*) | -1.253 | -3.627 | 0.683 |
|  |  | *b* | 3.406 | 1.202 | 6.420 |
|  |  | *Σ* | 2.237 | 0.015 | 9.495 |
| Presence*_i_* ~ *a* + *b* . fruit_prevalent*_i_* + *Σ* | 0.503 | intercept (*a*) | -1.096 | -3.192 | 0.517 |
|  |  | *b* | 3.662 | 1.849 | 6.760 |
|  |  | *Σ* | 1.940 | 0.017 | 8.640 |
| Presence*_i_* ~ *a* + *b* . insect_prevalent*_i_* + *Σ* | 0.140 | intercept *(a)* | 0.848 | -1.595 | 2.994 |
|  |  | *b* | -1.686 | -3.658 | 0.059 |
|  |  | *Σ* | 2.561 | 0.058 | 9.552 |
| Presence*_i_* ~ *a* + *b* . cave_roost*_i_* + *Σ* | 0.002 | intercept *(a)* | -0.178 | -3.157 | 1.896 |
|  |  | *b* | -0.255 | -1.848 | 1.809 |
|  |  | *Σ* | 3.256 | 0.222 | 10.795 |

**Table S8**

Summary of Bayesian regression models of the ln-transformed density of long-wave cones (plus one) as a function of whether or not fruit is prevalent in the diet, or whether or not species has S-cones. The estimated sample size>200 in every case. *R^2^*, variance explained by sample-wide factors; *Σ_j_*, species-specific phylogenetic effect for species *j*.

| **formula** | ***R^2^*** | **parameter** | **median** | **2.5%** | **97.5%** |
| --- | --- | --- | --- | --- | --- |
| ln(density)*_i_* ~ *a* + *b* . fruit_prevalent*_j_* + *Σ_j_* | 0.000 | intercept (*a*) | 8.42 | 8.00 | 8.84 |
|  |  | *b* | 0.22 | -0.21 | 0.66 |
|  |  | *Σ_j_* | 0.14 | 0.04 | 0.46 |
| ln(density)*_i_* ~ *a* + *b* . S-cones_present*_j_* + *Σ_j_* | 0.240 | intercept (*a*) | 8.28 | 7.95 | 8.63 |
|  |  | *b* | 0.43 | 0.09 | 0.78 |
|  |  | *Σ_j_* | 0.07 | 0.000 | 0.51 |
